# Molecular subgroups of T-cell acute lymphoblastic leukemia in adults treated according to pediatric-based GMALL protocols

**DOI:** 10.1038/s41375-024-02264-0

**Published:** 2024-05-14

**Authors:** Martin Neumann, Thomas Beder, Lorenz Bastian, Sonja Hänzelmann, Miriam Bultmann, Nadine Wolgast, Alina Hartmann, Heiko Trautmann, Jutta Ortiz-Tanchez, Cornelia Schlee, Michael Schroeder, Lars Fransecky, Sebastian Vosberg, Walter Fiedler, Nael Alakel, Lisa Heberling, Mustafa Kondakci, Michael Starck, Stefan Schwartz, Simon Raffel, Carsten Müller-Tidow, Folker Schneller, Albrecht Reichle, Thomas Burmeister, Philipp A. Greif, Monika Brüggemann, Nicola Gökbuget, Claudia D. Baldus

**Affiliations:** 1 Medical Department II, Hematology and Oncology, University Hospital Schleswig-Holstein, Kiel, Germany; 2 Clinical Research Unit ‘CATCH ALL’ (KFO 5010/1), Kiel, Germany; 3 University Cancer Center Schleswig-Holstein (UCCSH), University Hospital Schleswig-Holstein, Kiel and Lübeck, Germany; 4 Department of Hematology, Oncology and Tumor Immunology, Charité – Universitätsmedizin Berlin, Campus Benjamin Franklin, Berlin, Germany; 5 Department of Internal Medicine III, University Hospital LMU Munich, Munich, Germany; 6 German Cancer Consortium (DKTK), partner site Munich, Munich, Germany; 7 German Cancer Research Center (DKFZ), Heidelberg, Germany; 8 Medical Department II, Hematology and Oncology, University Hospital Hamburg Eppendorf, Hamburg, Germany; 9 Medical Department I, Carl Gustav Carus University Hospital Dresden, Dresden, Germany; 10 Department of Hematology, Oncology and Clinical Immunology, University Hospital Düsseldorf, Düsseldorf, Germany; 11 Medical Department I, Hospital München-Schwabing, Schwabing, Germany; 12 Medical Department V, University Hospital Heidelberg, Heidelberg, Germany; 13 Medical Department III, Klinikum rechts der Isar, Technical University Munich, Munich, Germany; 14 Department of Internal Medicine III, Hematology and Oncology, University Hospital Regensburg, Regensburg, Germany; 15 Department of Hematology, Oncology and Tumor Immunology, Charité – Universitätsmedizin Berlin, Campus Virchow-Klinikum, Berlin, Germany; 16 Department of Medicine II, Hematology/Oncology, University Hospital, Goethe University, Frankfurt/M, Germany

**Keywords:** Acute lymphocytic leukaemia, Cancer epigenetics

## Abstract

In contrast to B-cell precursor acute lymphoblastic leukemia (ALL), molecular subgroups are less well defined in T-lineage ALL. Comprehensive studies on molecular T-ALL subgroups have been predominantly performed in pediatric ALL patients. Currently, molecular characteristics are rarely considered for risk stratification. Herein, we present a homogenously treated cohort of 230 adult T-ALL patients characterized on transcriptome, and partly on DNA methylation and gene mutation level in correlation with clinical outcome. We identified nine molecular subgroups based on aberrant oncogene expression correlating to four distinct DNA methylation patterns. The subgroup distribution differed from reported pediatric T-ALL cohorts with higher frequencies of prognostic unfavorable subgroups like HOXA or LYL1/LMO2. A small subset (3%) of HOXA adult T-ALL patients revealed restricted expression of posterior *HOX* genes with aberrant activation of lncRNA HOTTIP. With respect to outcome, TLX1 (*n* = 44) and NKX2-1 (*n* = 4) had an exceptionally favorable 3-year overall survival (3y-OS) of 94%. Within thymic T-ALL, the non TLX1 patients had an inferior but still good prognosis. To our knowledge this is the largest cohort of adult T-ALL patients characterized by transcriptome sequencing with meaningful clinical follow-up. Risk classification based on molecular subgroups might emerge and contribute to improvements in outcome.

## Introduction

T-cell acute lymphoblastic leukemia (T-ALL) is an aggressive disease, which accounts for approximately 25% of all adult ALL patients. Over the last decades outcome has improved and is comparable to B-cell precursor (BCP-) ALL. Inferior outcomes are reported for adult patients with immature T-ALL [1, 2]. Whereas intensive pediatric-based chemotherapy with or without allogeneic stem cell transplantation (alloSCT) is effective, targeted therapies including an absence of used immunotherapies (monoclonal or bispecific antibodies or CAR-T-cell therapies) are lacking and thus options for high-risk subtypes and poor responders are limited.

Leukemogenesis in T-ALL is orchestrated by the overexpression of specific oncogenes [3]. Aberrant expression of oncogenes often rests upon structural aberrations (chromosomal translocations, copy number variations, point mutations) leading to overexpression using different promoters or enhancer hijacking directly or by changing epigenetic regulation of above mentioned T-ALL specific oncogenes [4]. Up to 10 molecular subgroups based on oncogene overexpression and/or defined genomic alterations have been described in T-ALL patients, preferentially determined in pediatric cohorts [5, 6]. Mutually exclusive affected genes include the transcription factors *TAL1* [7, 8], *TAL2* [9], *TLX1* [10], *TLX3* [11], and *NKX2*-1 [12], the homeobox HOXA genes and LIM-only domain genes *LMO1*/2. Early immature T-ALL is a more heterogeneous subgroup with overexpression of *LYL1* and *MEF2c* as a common feature [12, 13].

Until now molecular subgroup allocation of T-ALL is rarely considered for clinical risk stratification. Clinical decision-making including stratification to alloSCT is based on the immunophenotype, selected molecular aberrations (*NOTCH1, RAS, PTEN*) and minimal residual disease (MRD) as the main prognostic factor [14]. In the German Multicenter Study Group for Adult ALL (GMALL) protocols patients with early and mature T-ALL or molecular failure after first consolidation are regarded as high-risk [15].

Within the current WHO classification the early T-cell precursor (ETP) ALL is the only defined sub-entity of T-ALL [16]. Anticipating the molecular heterogenetic of T-ALL, ETP-ALL definition is yet based on surface antigens reflecting the gene expression status of physiologic early thymic progenitors [17]. In the International Consensus Classification of Acute Leukemias [18] molecularly defined provisional entities are outlined. However, in contrast to BCP-ALL, molecular subgroups are limited and less well defined [19].

Current results suggest that the distribution of molecular subgroups clearly differs across age groups [6]. As comprehensive studies in adult T-ALL patients are lacking, additional data are warranted to establish a robust molecular subgroup allocation with correlation to clinical parameters. Here, we investigated a large cohort of 230 adult T-ALL patients homogenously treated with contemporary protocols examining molecular levels of expression, DNA methylation, and DNA sequence alterations to allocate molecular subgroup profiles and assign the potential prognostic relevance.

## Patients and methods

### Patient cohort

We analyzed 230 adult T-ALL patients treated according to pediatric-inspired GMALL study protocols (2006-21). All patients were registered in prospective studies or the standardized population-based registry of the German Multicenter Study Group for Adult ALL (GMALL) ([2, 20, 21]. All 230 patients had available material from peripheral blood or bone marrow at first diagnosis with at least 20% blasts of all cells in the samples. For analysis of methylation analysis and targeted DNA sequencing, we demanded 60% blasts in the investigated samples. Clinical characteristics including age, sex, and immunophenotype are summarized in Supplementary Table S1. For n = 215 patients clinical data was available. All patients participating in the GMALL studies provided written informed consent, as required by the Declaration of Helsinki. The studies were approved by the ethics board of the Johann Wolfgang von Goethe University in Frankfurt/Main, Germany. Additionally, the protocol was approved by the respective committees at all participating institutes. Immunophenotyping was carried out as previously described [22, 23]. T-lineage leukemia was subclassified into early T-ALL (cyCD3+, CD7+, CD5+/−, CD2−, sCD3−, CD4−/+, CD8−/+, CD1a− or cyCD3+, CD7+, CD5−, CD2+, sCD3−, CD4−, CD8−, CD1a−), thymic T-ALL (cyCD3+, CD7+, CD5+/−, CD2+/−, sCD3+/−, CD4+, CD8+, CD1a+), and mature T-ALL (cyCD3+, CD7+, CD5+, CD2+, sCD3+/−, CD4+/−, CD8+/−, CD1a−). ETP-ALL was defined by weak or absent CD5 expression (<25%) and co-expression of myeloid or stem cell markers (CD 13, CD33, CD34, CD117, and/or HLA-DR). MRD was centrally analyzed in the GMALL MRD reference laboratory in Kiel using real-time quantitative (RQ)-PCR of clonal immunoglobulin and T-cell receptor rearrangements. RQ-PCR data were interpreted according to EuroMRD guidelines.

### RNAseq

RNAseq was performed in all 230 samples. RNAseq was done with polyA-enriched library protocols from bone marrow aspirates using the TruSeq RNA Library Prep kit (Illumina©, San Diego) for stranded mRNA. We sequenced the libraries on Illumina HiSeq2000 or NovaSeq platforms with 2 × 100-paired-end reads. On average, we achieved around 40 million reads per sample. We stored our data in the EGA archive box EGA50000000202. The analyses were performed as previously described [23]. Samples with RIN < 6 were excluded from sequencing and quality control on raw reads was performed using FastQC (S. Andrews, Babraham Bioinformatics). Raw reads were aligned to the human genome (GRCh38.p13, Ensembl annotations version 94) using STAR aligner version 2.7.9a [24]. Resulting gene counts were normalized using variant stabilization transformation and gene expression was analyzed using the R package DESeq2 version 1.32.0. Fusion transcripts were called from raw RNAseq reads using FusionCatcher version 1.33 and Ensembl human genome annotation version 104. Resulting gene fusions were filtered using a manually curated list of fusion breakpoints recurrent within the present cohort and/or described in the literature as driver fusions for ALL. Integrative Genomics Browser (IGV) version 2.4.19 [25] was used to visualize the results.

### Subtype classification

Machine learning T-ALL subtype classification was based on fpkm values from two cohorts i.e. *n* = 230 adult GMALL samples presented in this work and *n* = 264 pediatric T-ALL cases from St Jude published by Liu et al. [5]. Training was performed on 17,558 genes found in both datasets and *n* = 169 GMALL as well as *n* = 241 St Jude samples that could be assigned to a T-ALL subtype based on oncogene expression and gene fusion detection (Supplementary Fig. S1). Classifiers were trained on GMALL and St Jude datasets separately in a 10-fold randomized stratified cross-validation (CV) scheme, to test generalizability of the individual models. In short, feature selection, hyper-parameter tuning and training of the classifiers was done on 90% of the data. Ten percent of the data was exclusively used for testing the performance, thus leaving this data completely untouched by the machines during training. For feature selection, we applied least absolute shrinkage and selection operator (LASSO) regression with alpha parameter of 1. LASSO [26] was run in an internal 10-fold cross-validation with “type.measure = deviance” and “family = multinomial” logistic regression using the cv.glmnet function of the glmnet R package [27]. Classifier training was performed using Random Forest as implemented in “rf” in caret [28] with repeated CV or Leave-one-out CV.

### Mutational analysis

In 84 of adult T-ALL patients (Supplementary Table S2), we investigated the mutation status of 206 genes by targeted DNA sequencing (Supplementary Table S3). We constructed libraries from genomic DNA, which were labeled by barcode indices (length: 6 bp). Customized biotinylated RNA oligo pools (SureSelect, Agilent) were used to hybridize the target regions comprising the 206 selected genes. We used the Illumina HiSeq2000 platform with 100 bp paired-end sequencing and achieved in average 800 reads per base. Our analysis pipeline was previously described [29]. In addition, we investigated all samples of the TAL1, LMO1 subgroup and unclassified samples (in total 78 samples) for mutations in the TAL1 enhancer region by Sanger sequencing.

### DNA methylation

For assessment of the methylation status in addition to the mutational status of 84 T-ALL samples, we used the Infinium® HumanMethylation450 BeadChip platform. For analysis of the Infinium® HumanMethylation450 BeadChip data, we used in R software the dasen function (wateRmelon package12,13). dbSNP-related CpGs with MAF > 0.01 were filtered out and beta values of methylation in sex-related positions CpGs were removed. For identifying subgroups principal component analysis with the top 2000 most variable CpGs was performed. Differentially methylated genes and regions were determined in R software to apply 1000 permutations with the Bumphunter14 algorithm (see also [30]).

### Statistical analysis

Differences in the clinical characteristics were tested by the Pearson *χ*^2^ test, resp. Fisher test. Differences in the mutation rate were analyzed by the Pearson *χ*^2^ test. For all tests, a *P* value < 0.05 (two-sided) was considered to indicate a significant difference. Comparions regarding expression of single genes were performed with Mann–Whitney *U*-test. Multiple testing was corrected using the false discovery rate (FDR) adjustment based on the Benjamini-Hochberg correction method. All calculations were performed using the SPSS software version 17 (SPSS Inc., Chicago, IL, USA) and GraphPad Prism® software version 5 (GraphPad Software Inc., La Jolla, CA, USA).

## Results

We investigated 230 adult T-ALL patients enrolled in GMALL registries and trials with a median age of 32 years (range 17-83 years); of these 11 patients (5%) were older than 55 years. 166 (72%) patients were male: 107 (46.5%) patients showed an immunophenotype of a thymic T-ALL, 43 (18.7%) of a mature T-ALL, and 60 (26.1%) of an early T-ALL, for 20 patients immunophenotype was missing (Table 1).

**Table 1:** Cohort characterization.

			Immunophenotype		
		Total	Thymic	Mature	Early
		230	107	43	60
Age (years)					
	≤55	219 (95%)	105 (98%)	40 (93%)	56 (93%)
	>55	11 (5%)	2 (2%)	3 (7%)	4 (7%)
Sex					
	Female	64 (28%)	33 (31%)	9 (21%)	18 (30%)
	Male	166 (72%)	74 (69%)	34 (79%)	42 (70%)
ETP					
	Yes	21 (10%)	0 (0%)	2 (5%)	19 (32%)
	No	189 (90%)	107 (100%)	41 (95%)	41 (68%)
Methylation cluster					
	M1	25 (11%)	7 (7%)	10 (23%)	8 (13%)
	M2	20 (9%)	12 (11%)	8 (19%)	0 (0%)
	M3	25 (11%)	20 (19%)	3 (7%)	1 (2%)
	M4	12 (5%)	10 (9%)	1 (2%)	1 (2%)
Molecular subgroup					
	LMO1	15 (7%)	9 (8%)	6 (14%)	0 (0%)
	TLX3	26 (11%)	10 (9%)	6 (14%)	7 (12%)
	TLX1	44 (19%)	42 (39%)	0 (0%)	0 (0%)
	LYL1LMO2	32 (14%)	3 (3%)	10 (23%)	15 (25%)
	HOXA	50 (22%)	19 (18%)	4 (9%)	23 (38%)
	TAL1LMO	30 (13%)	17 (16%)	9 (21%)	2 (3%)
	HOXA13	7 (3%)	0 (0%)	1 (2%)	6 (10%)
	NKX2	4 (2%)	3 (3%)	1 (2%)	0 (0%)
	TAL2	1 (0%)	0 (0%)	0 (0%)	0 (0%)

The table shows the frequencies for age, sex, ETP status, molecular subgroup, and methylation cluster in the overall cohort and according to their immunophenotype. Immunophenotype is known for 210 out of the 230 T-ALL patients.

### Transcriptome sequencing reveals nine molecular subgroups in adult T-ALL

**Fig. 1:**
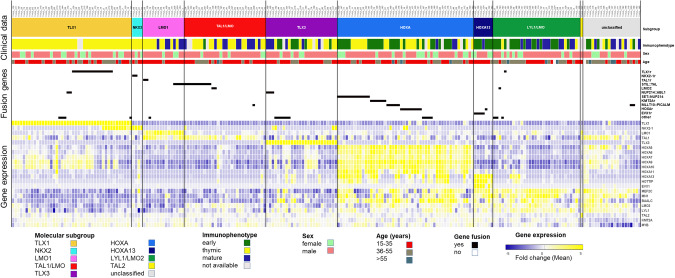
Molecular subgroups in adult T-ALL. Classification of 230 adult T-ALL patients into molecular subgroups based on RNAseq. Clinical data, fusions genes, and oncogene expression are shown.

For the molecular subgroup assignment, we built a class prediction model based on two cohorts. One part of the investigated cohort served as an internal reference cohort, consisting of 169 of the 230 adult T-ALL GMALL samples. This cohort was previously assigned based on oncogene expression and served as a training cohort. In addition, an external cohort of pediatric T-ALL cases (*n* = 241) published by Liu and colleagues was used as a second reference cohort [5]. We were able to assigned the predicted molecular subgroup in 175 samples (76%) based on overlapping predictions using the internal reference cohort and the external reference cohort. Of the remaining 55 samples, we assigned additional 11 samples based on their specific fusion genes and 23 samples manually classified by a clear oncogene overexpression and similarity of gene expression profiles to those of already defined samples (Supplementary Fig. S1). Twenty-one of 230 T-ALL samples remained unclassified, representing 9.1% of the total cohort (Fig. 1).

The largest molecular subgroup in adult T-ALL was the HOXA subgroup defined by overexpression of *HOXA* genes in 57 samples (24.7%) (Fig. 1, Supplementary Table S4). In the majority of these cases early stem cell markers like *MEF2C* and *BAALC* were co-overexpressed compared to the expression in subgroups TLX1, TAL1/LMO, TLX3 and LMO1 (*p* < 0.001). The expression of stem cell genes is also a hallmark of the immature subgroup LYL1/LMO2, covering 32 samples (13.9%) and lacking expression of HOXA cluster genes. Samples with *TLX1* overexpression comprised 44 samples (19.1%) harboring the defining *TLX1* overexpression. Eleven of these TLX1 samples expressed *NKX2*, similar to the NKX2 subgroup comprising 4 samples (1.7%) lacking *TLX1* overexpression. Of the TAL1/LMO subgroup (in total 45 samples), 15 samples (6.5%) had an *LMO1* overexpression, while the remaining 30 samples (13%) were lacking *LMO1* expression. Consecutively, we defined these two groups LMO1 and TAL1/LMO as separate entities. While samples of the TLX1 and TLX3 subgroup showed some HOXA expression, TAL1/LMO and LMO1 cases lacked overexpression of *HOXA* genes. *TLX3* overexpression was found in 26 samples (11.3%) defining the TLX3 subgroup with absence of expression of *TLX3* in all other subgroups. We identified a single sample with *TAL2* overexpression. Notably, no fusion or overexpression of *SPI1* was detected in our cohort.

**Fig. 2:**
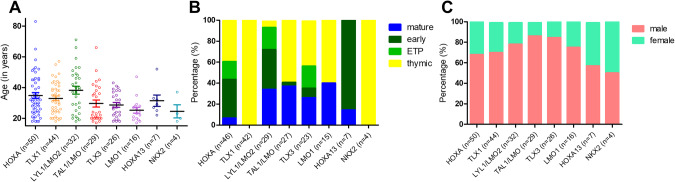
Clinical features of molecular subgroups in adult T-ALL. **A** Age distribution according to the molecular subtypes. **B** Immunophenotype according to molecular subgroups. Number of samples varies from (**A**) and (**B**) due to missing immunophenotypes for 15 samples. **C** Gender distribution in the molecular subgroups.

The average age ranged from 24 years to 38 years across the molecular cohort and displayed highest in LYL1/LMO2 with a median of 39 years and lowest in NKX2 with a median of 21.5 years (Fig. 2A). In our cohort, age distribution revealed younger patients with TAL1/LMO overexpression (16-25 years: 35% versus >35 years: 3%; *p* = 0.001) and more LYL1/LMO2 and HOXA overexpression among older patients (16–25 years: 23% vs. >35 years: 40%; n.s.).

The subgroups indicated a clear correlation with immunophenotype reflecting the T-cell development stage (Fig. 2B). All patients in the TLX1 and NKX2 subgroup revealed a thymic immunophenotype (*p* < 0.001). LYL1/LMO2 (*p* = 0.017) and HOXA (*p* = 0.05) were characterized by a more immature phenotype, showing surface expression of antigens typical for an early T-ALL in 50% of samples. TLX3 was not associated with a specific immunophenotype in this cohort with 23 samples (early: *n* = 7, thymic: *n* = 10, mature: *n* = 6, n.s.). On the other hand, TAL1/LMO was associated with more mature T-cell development stages, reflected by a thymic and mature T-ALL immunophenotype (*p* = 0.002). Only a single TAL1/LMO cases had an early T-ALL immunophenotype. Twenty-three (34%) of the 67 early T-ALL samples showed an ETP-ALL immunophenotype. By note, of 23 samples with an ETP-ALL immunophenotype 11 belong to the HOXA molecular subgroups, six were in LYL1/LMO2, five in TLX3, and one could not be classified. Regarding sex distribution, no significant differences were observed between molecular subgroups (Fig. 2C).

### HOXA13 as distinct T-ALL subgroup

**Fig. 3:**
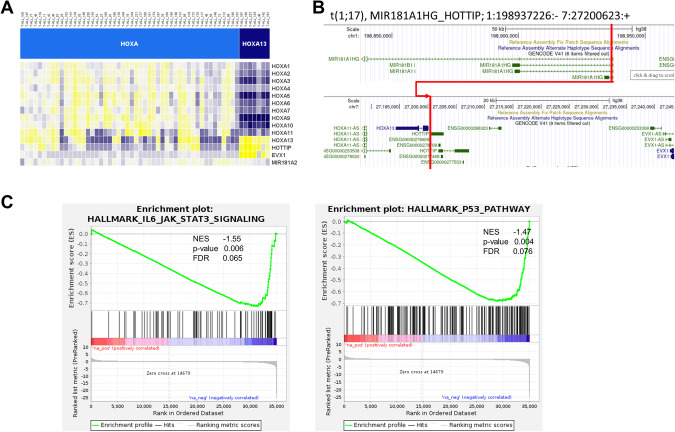
HOXA13 cluster with recurrent mir181A1HG_HOTTIP fusion. **A** Expression of posterior HOXA genes in HOXA13 cluster. **B** Fusion gene mir181A1HG_HOTTIP with the 5′ fusion partner breakpoint after exon partner and full usage of 3′ fusion partner HOTTIP. **C** Differentially expressed genes between HOXA13 and HOXA cluster. GSEA enrichment analysis for JAK/STAT and TP53 pathway compared between HOXA13 and HOXA.

Seven patients (3% of the total cohort) showed a distinct overexpression of *HOXA13* with associated overexpression of the lncRNA *HOTTIP* and *EVX1* (Figs. 1 and 3A). Notably, in these samples the anterior *HOXA* genes were not expressed on transcript level. In five out of the seven samples, fusion genes targeting the posterior HOXA complex were identified. Three cases with recurrent *MIR181A1HG::HOTTIP* were caused by a chromosomal t(1;7) translocation with subsequent overexpression of lncRNA *HOTTIP* (Fig. 3B). In two distinct detected fusion genes in HOXA13 cluster, *HOXA13* and *EVX1* were involved (Supplementary Table S5). All 7 samples depicted an early immunophenotype reflected by missing sCD3 expression. Four additional samples showed an exclusive *HOXA13* overexpression among the *HOXA* genes, but were classified in the LYL1/LMO2 subgroup. All four samples were lacking *EVX1* overexpression.

HOXA13 cases showed a distinct gene expression profile compared to the HOXA samples with the majority of differentially expressed genes being downregulated in HOXA13 cluster, including expected downregulation of anterior HOXA genes (Supplementary Table S6). In gene set enrichment analyses of KEGG and Hallmark of cancer gene lists, we identified 88 differentially regulated gene sets (FDR < 0.1, Supplementary Table S7). Among those, JAK-STAT-signaling and TP53 pathway were altered in HOXA13 compared to HOXA (Fig. 3C), which have been associated with poor prognosis (TP53) and association to the unfavorable immunophenotype of early T-ALL (JAK/STAT mutations).

### Underlying fusion genes drive T-ALL leukemogenesis

In 106 of the 230 T-ALL samples we detected underlying fusion genes in T-ALL (Fig. 1, Supplementary Table S5). The most frequent rearrangements in our cohort were *STIL::TAL1* (*n* = 14) and *TLX1*-rearrangements (*n* = 15). *SET::NUP214* occurred in 12 samples and was associated with the HOXA subgroup. Fusions of NKX2 were a rare events in adult T-ALL patients with a rate of 1% in the overall cohort. Further recurrent gene fusions affected among others *KMT2A* (*n* = 6), *MLLT10* (*n* = 5) or *LMO2* (*n* = 2) (Supplementary Table S5). In LYL1/LMO2 only few rearrangements were found, underlying the stem cell character of LYL1/LMO2 with similarities to acute myeloid leukemia (AML). Notably, all detected fusions confirmed subgroup assignment based on gene expression.

### Mutational spectrum of T-ALL according to molecular subgroups

The different composition of molecular subgroups was reflected by the mutational spectrum of adult T-ALL. We investigated full protein coding sequence of 206 leukemia-associated genes (Supplementary Table S3). Twenty-two of these genes were affected at least in 3% of all samples. The most frequently altered gene was *NOTCH1* with 42 out of 83 (51%) mutated samples followed by *PHF6* (31/83 samples; 37%) and *PTEN* (15/83 samples; 18%). The small sample sizes in the molecular subgroups made firm comparisons between them difficult (see Supplementary Tables S8 and S9). However, a trend indicated that the TLX1 subgroup has a higher rate of *NOTCH1* mutations, with 15 out of 21 samples (71%) being mutated, compared to the more immature molecular subgroups such as LYL1/LMO2 (5/12; 42% *NOTCH1* mutated) or HOXA (4/12; 33% *NOTCH1* mutated; not significant). On the other hand, genes affecting the JAK/STAT pathway (*JAK1*, *JAK3*, *STAT5B*, *SH2B3*, *IL7R*), were more frequently affected in the HOXA (5/12, 42%) and LYL1/LMO (7/12, 58%) subgroups compared to the TLX1 subgroup (6/21, 29%). However, these differences remained descriptive and did not reach statistical significance.

In addition, for 78 samples (all TAL1 and LMO1 samples and unclassified samples), we investigated the mutational status of the enhancer region of TAL1 with a previously reported mutation in the non-protein-coding region [31]. We only found two mutations (2/78, 3%) in the investigated cohort, both in samples of the molecular subgroup TAL1. As it is unlikely to find enhancer mutations in non-TAL1 overexpressing samples, the rate of 1% referring to the total adult T-ALL cohort seems lower than reported for pediatric patients.

### Methylation patterns revealed hypomethylation in STIL::TAL1 subgroup

**Fig. 4:**
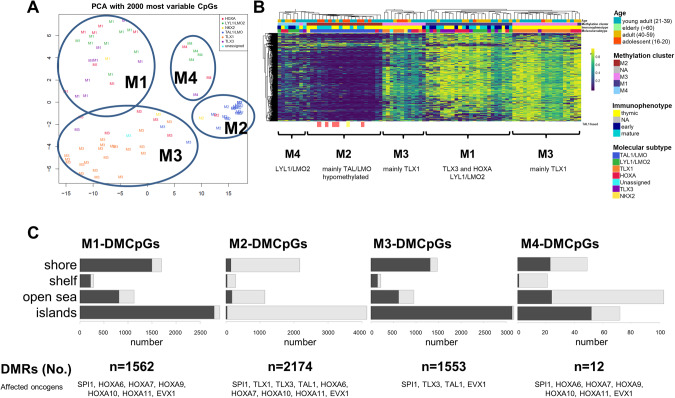
Methylation profile of adult T-ALL samples. **A** Principal component analysis of 84 T-ALL samples based on their methylation status using the 2000 most variant CpGs resulting in four stable clusters (M1–M4). **B** Methylation clusters showed a high concordance with molecular subgroups and immunophenotype. Methylation level of the 2000 most variant CpGs are presented with a clear hypomethylation for cluster M2. Below the graph most prominent molecular subgroups in the methylation clusters are depicted. Samples with an underlying TAL1 fusion are marked. **C** Number of differentially methylated regions and their methylation status according to the four methylation subgroups. Genes in the differentially methylated regions are reflecting driving oncogenes of molecular T-ALL subgroups.

In 84 investigated samples, four clusters were identified based on unsupervised clustering, reflecting molecular subgroups on DNA methylation level (Fig. 4A, Supplementary Fig. S2). Cluster M1 consisted of 25 samples, comprising 8 out of 10 TLX3 samples, 8 LYL1/LMO2 samples and 8 HOXA samples. Only one NKX-1 sample was included. In terms of immunophenotype, cluster M1 reflected a more heterogeneous picture comprising high-risk features according to GMALL protocols (10 mature T-ALL samples and 8 out of 12 samples with an early immunophenotype; Fig. 4B). Cluster M2 included 20 samples, nearly exclusively TAL1/LMO samples (*n* = 18). Vice versa 18 of the 20 TAL1/LMO2 samples were assigned into the M2 cluster, including all samples with a TAL1 fusion (*STIL::TAL1* fusion *n* = 5; *TCF7::TAL1* fusion *n* = 1) showing a significant global hypomethylation in CpG islands (Fig. 4C). The largest cluster, M3, included 30 samples. Remarkably, 29 samples in this cluster showed a thymic immunophenotype and all TLX1 samples (*n* = 20) demonstrated a DNA methylation pattern representative of cluster M3. In the remaining samples of cluster M3 we found 6 HOXA samples, and one TLX3, NKX2-1, and TAL1/LMO sample each. The fourth cluster M4 revealed to be the smallest with only 7 samples (4 LYL1/LMO2, 2 HOXA and 1 TLX3 samples). Taken together, DNA methylation profiling enabled a robust subclassification of adult T-ALL samples. TLX1 and TAL1/LMO subgroups were characterized by a homogenous common DNA methylation pattern reflected by the assignment in the same methylation cluster (TLX1: 20/20 in M3, TAL1/LMO: 18/20 in M2), separating both subgroups from other molecular subgroups. On the other hand, TLX3, HOXA and LYL1/LMO2 had common DNA methylation patterns in cluster M1 and M4, associated with an early immunophenotype (early T-ALL: 17/19 in M1 and M4 vs. 2/19 in M2 and M3, *p* < 0.0001). Among the differentially methylated regions (DMRs) for each of the four clusters, defining oncogenes of the corresponding molecular subgroups were found (Fig. 4C, Supplementary Tables S10 and S11). The association of mutational events with the methylation clusters revealed a high rate of *NOTCH1* mutations in cluster M3 and an increased rate of mutations in the JAK/STAT pathway and epigenetic regulators in M1 and M4 clusters (data not shown).

### MRD response according to molecular subgroup in adult T-ALL

With respect to response, 95 (68%) patients with a molecular marker achieved a molecular CR (MolCR) after consolidation I, 25 (18%) patients showed an intermediate molecular response (IntMR) and 19 (14%) failed molecular remission (MolFail) (Table 2). In the subgroup of thymic T-ALL, 65 patients (79%) achieved MolCR, 9 (11%) patients IntMR and 8 (10%) patients failed. Between the molecular subgroups, MRD after consolidation I differed significantly. Noteworthy, 37/39 (95%) of TLX1 patients had a MolCR with only two patients in TLX1 subgroup with a MolFail. On the other side, in LYL1/LMO2 only 3/9 (33%) got into MolCR. Similarly, TLX3 and TAL1/LMO subgroup showed a significantly worse response with a rate of only 50% of MolCR (Table 1). HOXA subgroups showed an intermediate response with a rate of 65% (20/37 patients) of MolCR. These response rates of the molecular subgroups are similar in patients with a thymic immunophenotype despite their favorable CD1a expression: MolCR rate in HOXA 73%, in TLX3 60% and in TAL1/LMO 40% with only limited number of patients. Thymic T-ALL patients with a TLX1 or NKX2 profile showed excellent MRD response (95% resp. 100% of MolCR; Table 2).

**Table 2:** Minimal residual disease in molecular subgroups.

MRD according to molecular subgroups				
Subgroup	Number of samples	MolCR *n* = (%)	MoIMR *n* = (%)	MolFail *n* = (%)
	**Overall cohort (*n* = 196)**			
TLX1	39	37 (95%)	0 (0%)	2 (5%)
HOXA	37	24 (64%)	9 (24%)	4 (11%)
TAL1/LMO	22	11 (50%)	6 (27%)	5 (23%)
TLX3	18	9 (50%)	5 (23%)	4 (22%)
LMO1	11	9 (82%)	0 (0%)	2 (18%)
LYL1/LMO2	9	3 (33%)	4 (44%)	2 (22%)
NKX2	2	2 (100%)	0 (0%)	0 (0%)
TAL2	1	0 (0%)	1 (100%)	0 (0%)
HOXA13	0	0 (0%)	0 (0%)	0 (0%)
total	139	95 (68%)	25 (18%)	19 (14%)
	**Thymic T-ALL (*n*** = **99)**			
TLX1	39	37 (95%)	0 (0%)	2 (5%)
HOXA	15	11 (73%)	3 (20%)	1 (7%)
TAL1/LMO	10	4 (40%)	4 (40%)	2 (20%)
TLX3	10	6 (60%)	2 (20%)	2 (20%)
LMO1	6	5 (83%)	0 (0%)	1 (17%)
NKX2	2	2 (100%)	0 (0%)	0 (0%)
LYL1/LMO2	0	0 (0%)	0 (0%)	0 (0%)
total	82	65 (79%)	9 (11%)	8 (10%)

The minimal residual disease status after consolidation I according to molecular subgroups in T-ALL is displayed. For 57 samples with available clinic annotations minimal residual disease values are missing, this is the case for 17 samples in the thymic T-ALL. MolCR, negative with a minimum sensitivity of 10^−4^; MolMR, MRD positive, below 10^−4^ or not quantifiable; MolFail, MRD > 10^−4^.

### Favorable outcome in adult T-ALL is associated with TLX1, NKX2, and LMO1 subgroups

**Fig. 5:**
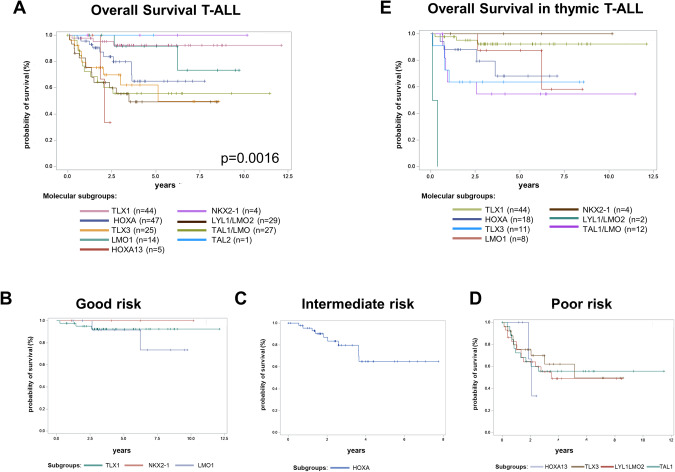
Overall survival for molecular subgroups in T-ALL. (**A**) Overall survival for the molecular subgroups in 196 adult T-ALL patients independent of their immunophenotype. Unassigned samples are not displayed. Colors for the different subgroups are shown below the figure. **B**-**D** Overall survival of adult T-ALL patients according to their molecular risk classification. Good risk group comprises 62 patients: TLX1 (*n* = 44), NKX2-1 (*n* = 4) and LMO1 (*n* = 14); intermediate risk group 47 patients: HOXA (*n* = 47); poor risk subgroup 76 patients: HOXA13 (*n* = 5), TLX3 (*n* = 25), LYL1/LMO2 (n = 29), TAL1/LMO2 (*n* = 27). **E** Overall survival for the molecular subgroups in 99 adult T-ALL patients with thymic immunophenotype. Unassigned samples are not displayed. Colors for the different subgroups are shown below the figure.

MRD response translated in OS with differences among the molecular subgroups. OS after three years differed significantly across the molecular subgroups in adult T-ALL (*p* = 0.0016, Fig. 5A). Among the larger T-ALL molecular subgroups, TLX1 patients (*n* = 44) showed an exceptional favorable 3y-OS of 92%. Although the sample size is too small to draw firm conclusions, in the small groups of NKX2-1 (*n* = 4) and TAL1 (*n* = 1), all patients were alive after three years. In addition, patients of the LMO1 subgroup (*n* = 14) had a very favorable 3y-OS of 92%. Patients of the HOXA (*n* = 47) and of the TLX3 (*n* = 25) subgroups with a 62% 3y-OS showed a slightly inferior prognosis (70% 3y-OS and 62% 3y-OS, respectively). In contrast, the subgroups LYL1/LMO2 (55% 3y-OS, *n* = 29), HOXA13 (33% 2y-OS, *n* = 5) and TAL1 (without LMO1 overexpression, 56% 3y-OS, *n* = 27) had a poorer prognosis.

Based on our cohort, we were able to classify T-ALL patients based on their molecular subgroups into good risk (TLX1, NKX2-1, LMO1), intermediate risk (HOXA), and poor risk (LYL1/LMO2, HOXA13, TAL1/LMO, TLX3). (Fig. 5 B-D). The favorable impact of TLX1 (92% 3y-OS, *n* = 44), LMO1 (89% 3y-OS, *n* = 8) and NKX2-1 (100% 3y-OS, *n* = 4) was not only found in the overall cohort but also within the already good risk subgroup of thymic T-ALL patients (Fig. 5E). In thymic T-ALL, fewer patients were assigned to the unfavorable subgroups of LYL1/LMO2 (0% 3y-OS, *n* = 2), TAL1 (55% 3y-OS, *n* = 12), TLX3 (64% 3y-OS, *n* = 11) and HOXA (79% 3y-OS, *n* = 13) compared to mature and early T-ALL. Although the relative incidences of these subgroups were lower, 45% of thymic T-ALL belonged to the HOXA, TLX3, TAL1 or LYL1/LMO2 subgroup. Therefore, a relevant percentage lacked the good prognosis. The above established molecular risk classification (Fig. 5 B–D) also allowed to stratify until now standard risk thymic T-ALL patients according to their molecular subgroup into distinct risk groups (good risk: 92% 3y-OS, intermediate risk 79% 3y-OS, poor risk 58% 3y-OS, *p* = 0.0022, Supplementary Fig. S3).

## Discussion

Classifying T-ALL on a molecular level remains challenging and thus molecular subgroups have not yet been incorporated into the classifications of WHO and only as provisional entities in the International Consensus Classification [16, 18]. Eight provisional subentities have been proposed in the International Consensus Classification [16] and a diagnostic approach using whole genome sequencing has been proposed [32]. Although it is obvious that dysregulations of certain oncogenes, partly based on common genetic alterations, drive specific T-ALL phenotypes, the exact definitions of subgroups are a matter of debate, in particular in adult patients.

Herein, we investigated a large cohort of 230 uniformly treated adult T-ALL patients by whole transcriptome analysis to identify a distinct distribution of molecular subgroups and their clinical impact. Remarkably, TAL1 was less frequent (19.5%) in adult T-ALL compared to published data of a cohort of pediatric, adolescent and young adult patients up to an age of 30 years (40%) [5], and SPI1 fusions were not detected in adult T-ALL. HOXA subgroup assignment was more frequent in adult T-ALL (24.5% vs. 13.7%) comprising samples with fusions for *KMT2A*, *MMLT10*, and *HOXA10*. This age distribution with higher frequencies for HOXA subgroup is in line with a higher rate of mutations in epigenetic regulators (most prominent *PHF6* and *DNMT3A*) and members of JAK/STAT pathway compared to published data [5, 6]. The frequencies for LYL1/LMO2 with 13.9% were also higher in our cohort, while we report only few NKX2-1 (1.7%) cases.

Alterations in non-coding regions have been frequently described in T-ALL, particularly in enhancer regions, including the TAL1, LMO1 and LMO2 genes [31, 33, 34]. We describe a low frequency of non-coding alterations in the TAL1 enhancer region in adult T-ALL. The extent to which this also applies to other regions of other genes remains to be investigated, as the present analyses in this paper are limited to the coding regions of the genes investigated, with the exception mentioned.

A more robust assignment to small subgroups with unique molecular phenotypes is possible with an increasing number of investigated samples as it was shown in BCP-ALL where the analyses of large cohorts (*n* > 3000) finally allowed a precise characterization of even very rare subgroups [23]. Until now, a concise assignment to a molecular subgroup in the absence of specific drivers or fusion genes remains challenging. In this context, rare subgroups like the HOXA13 cluster with a similar gene expression profile compared to the LYL1/LMO2 subgroup might be defined more precisely and especially the LYL1/LMO2 subgroup likely consists of several distinct subentities with distinct molecular drivers. One prominent example is the detection of BCL11B expressing early T-ALL subgroup [35]. Detection of TCR fusions is limited by RNAseq, thus several fusions involving especially *TLX1* or *TLX3* with a clear overexpression might be missed in our study. However, based on gene expression *TLX1* and *TLX3*-driven T-ALL could unequivocally be detected. Despite the described limitations, 91% of all samples could be robustly classified in our large homogenously treated adult T-ALL cohort.

Furthermore, the DNA methylation signature provides additional value to categorize T-ALL subgroups with similar underlying driver events [36, 37]. There is a significant overlap in classifying samples based on DNA methylation or gene expression, but the limited number of investigated methylation profiles is not sufficient for subclustering into eight subgroups. However, the presence of a very homogeneous methylation profile in TAL1/LMO, particularly in samples with *STIL::TAL1* fusions [38], contrasts with the much more heterogeneous picture seen in other subgroups like HOXA, highlighting the importance of investigating multiple layers of molecular regulation.

HOXA13 formed a unique subgroup, separated from the remaining HOXA cluster due to the sole expression of *HOXA13* in combination with the overexpression of *EVX1* and *lncRNA* HOTTIP. A recurrent fusion of *HOTTIP* with *MIR181A1HG* leads to the overexpression of *HOTTIP* and might be the initial event. Overexpression of long non-coding RNA (lncRNA) *HOTTIP* has been also described in AML [39, 40]. In normal tissues, *MIR181A1HG* has its highest expression in thymic cells. As the breakpoint occurs after exon 1, it is likely that ectopic expression of *HOTTIP* is caused by promoter activity of *MIR181A1HG*. In general, a dysregulated *HOXA13* expression has been connected to underlying *HOXA13* fusions resulting in altered 3D chromatin configuration as contributing factor to T-ALL leukemogenesis [41]. Thus, we could for the first time confirm HOXA13 as a high-risk subgroup in an adult T-ALL cohort.

Epigenetic effects of chromatin configuration are often controlled by genetic changes and/or imprinted in the cell of origin. Consistent with this, the global DNA methylation patterns reflect the molecular subgroup in high agreement. T-ALL samples characterized by a marked global hypomethylation (cluster M2) relate to a larger part of TAL1 samples with *STIL::TAL1* fusions. Also, other subgroups share common DNA methylation signatures underlining the close interplay of transcriptional activity with DNA methylation in both directions.

Patients with TLX1 revealed a molecular subgroup with extraordinarily good prognosis with a 3y-OS of 92%. All these samples showed a thymic immunophenotype with a high rate of *NOTCH1* mutations, which confirms the prognostic value of thymic T-ALL. The good, but less favorable outcome of TAL1 subgroup patients despite its favorable immunophenotype and in contrast to an excellent outcome in pediatric patients might be partly explained by the higher frequency of *PHF6* mutations. These mutations were also observed in pediatric patients, although very rare events, and were associated with an inferior outcome [42].

We were able to define a new molecular classification for the overall cohort of adult T-ALL based on the prognostic value of molecular subgroups into good risk (TLX1, NKX2-1, LMO1), intermediate risk (HOXA), and poor risk (LYL1/LMO2, TAL1/LMO, HOXA13, TLX3). This classification maintained its prognostic value also in the so far standard risk group of thymic T-ALL (3y-OS 84%) [2] and thus allowed to identify patients with higher risk despite the favorable thymic immunophenotype.

In conclusion, we investigated a large cohort of homogenously treated adult T-ALL patients and were able to define nine different subgroups with enrichment of the molecular subgroups HOXA and LYL1/LMO2 in adult T-ALL patients. These molecular subgroups showed distinct clinical features as well as close correlation to characteristic DNA methylation profiles. Furthermore, they differentiated with respect to the prognosis (i.e. overall survival). The analysis confirmed the feasibility of RNAseq to characterize T-ALL at first diagnosis and underlines the suggestion that RNAseq should be integrated into the standard diagnostic procedures to define molecular subgroups similar to BCP-ALL.

### Supplementary information

Supplementary legends

Supplemental Tables

Supplement Figure S1-3, Table S8

## Data Availability

The datasets generated during and analyzed during the current study are stored in the EGA archive box EGA50000000202.
