# Supplementary material for: Molecular subgroups of T-cell acute lymphoblastic leukemia in adults treated according to pediatric-based GMALL protocols
Source: Leukemia. 2024 May 14;38(6):1213–22. doi: 10.1038/s41375-024-02264-0 (PMC11147771; doi:10.1038/s41375-024-02264-0)
Supplement: Supplementary file 1 — Supplementary legends [file 41375_2024_2264_MOESM1_ESM.docx]

**Supplementary figure S1.** Schematic workflow for classification of 230 adult T-ALL patients.

**Supplementary figure S2.** UMAP plot of gene expression data using LASSO genes for each subgroup. (A) Samples are coloured according to their molecular subgroup. (B) Samples are coloured according to their methylation subgroup. (C) Samples are coloured according to their molecular subgroup, shapes represent the methylation subgroup.

**Supplementary Figure S3.** Overall survival in thymic T-ALL according molecular risk groups. Like in the overall cohort, low risk comprises TLX1, NKX2-1 and LMO1 subgroups; intermediate risk HOXA, and poor risk in thymic T-ALL comprises LYL1/LMO2, TLX1 and TAL1/LMO.

**Supplementary Table S1**. Characteristics and subgroup assignment of patient cohort.

**Supplementary Table S2**. Comparison of patient cohorts with and without methylation analyses.

**Supplementary Table S3.** Genes investigated in DANN sequencing anlyses.

**Supplementary Table S4**. Frequencies of molecular subgroups.

**Supplementary Table S5.** Complete list of detected fusion genes.

**Supplementary Table S6.** Differential gene expression of HOXA13 subgroup.

**Supplementary Table S7**. Gene set enrichment analysis for HOXA13 subgroup.

**Supplementary Table S8.** Mutations according molecular subgroups.
Mutation status for 204 leukemia associated genes in 83 adult T-ALL patients. The 22 most frequently affected genes known to be mutated in T-ALL are shown. A list of investigated genes could be found in Supplementary Table S3. The full list of all variants is depicted in Supplementary Table S9.

**Supplementary Table S9.** Complete list of called SNVs.

**Supplementary Table S10.** Differential methylation regions in methylation cluster.

**Supplementary Table S11**. Pathway analyses of differential methylation regions in methylation cluster.
