# Supplementary material for: Molecular subgroups of T-cell acute lymphoblastic leukemia in adults treated according to pediatric-based GMALL protocols
Source: Leukemia. 2024 May 14;38(6):1213–22. doi: 10.1038/s41375-024-02264-0 (PMC11147771; doi:10.1038/s41375-024-02264-0)
Supplement: Supplementary file 3 — Supplement Figure S1-3, Table S8 [file 41375_2024_2264_MOESM3_ESM.pdf]

Supplementary figure S1

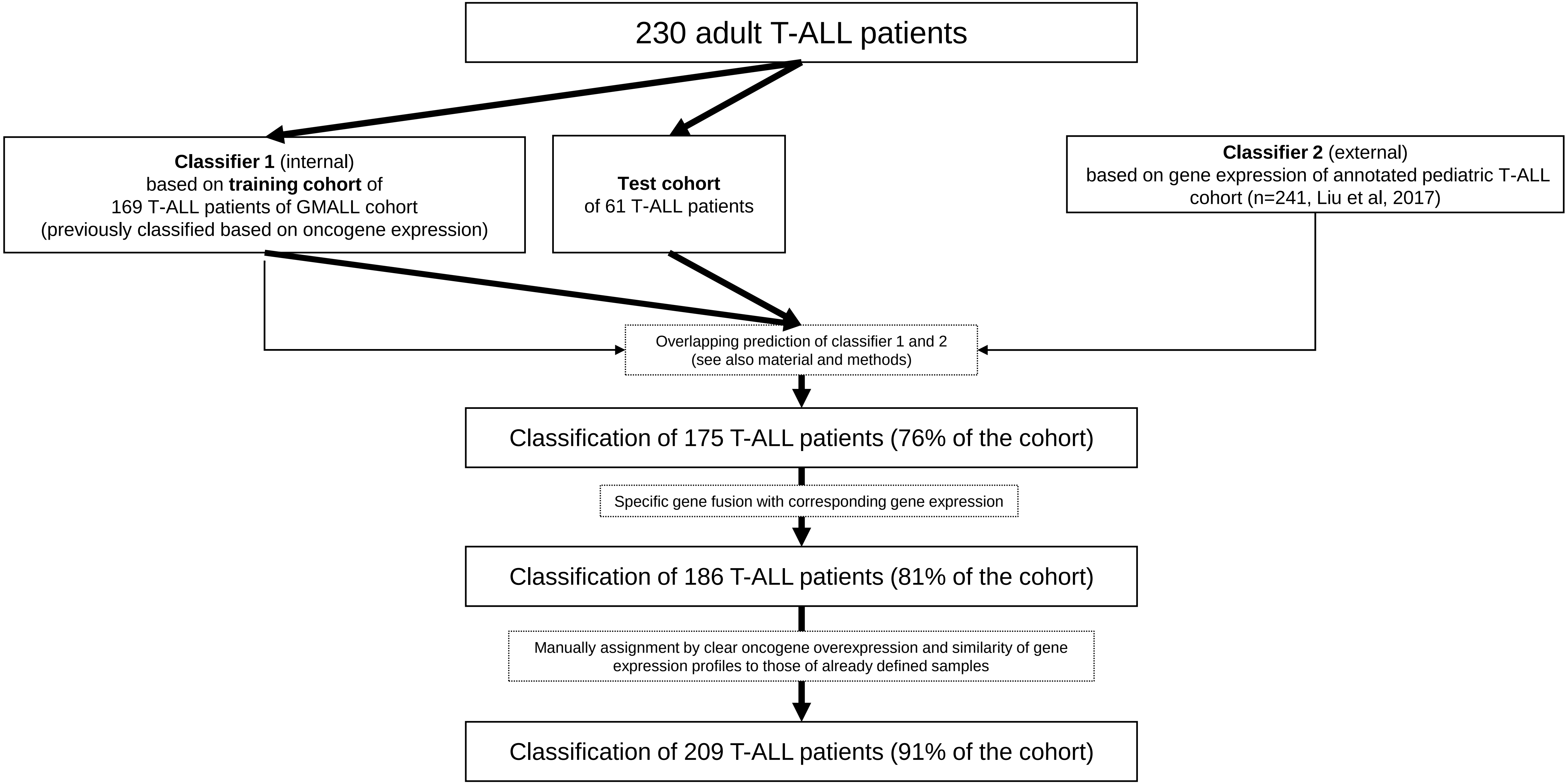

Supplementary figure S2

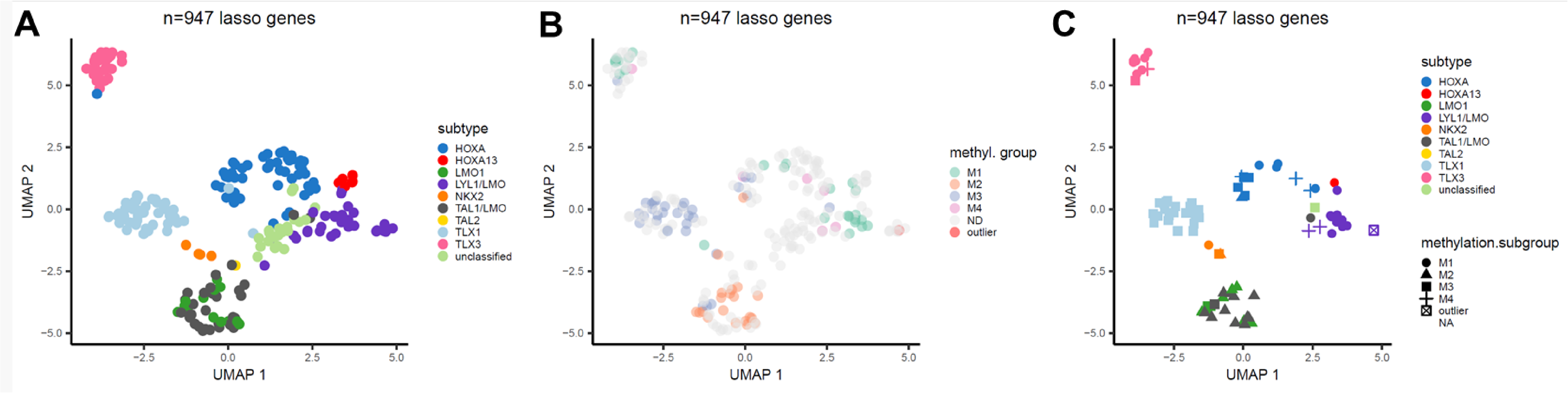

Supplementary figure S2 UMAP plot of gene expression data using LASSO genes for each subgroup. (A) Samples are coloured according to their molecular subgroup. (B) Samples are coloured according to their methylation subgroup. (C) Samples are coloured according to their molecular subgroup, shapes represent the methylation subgroup.

Overall survival in thymic T-ALL

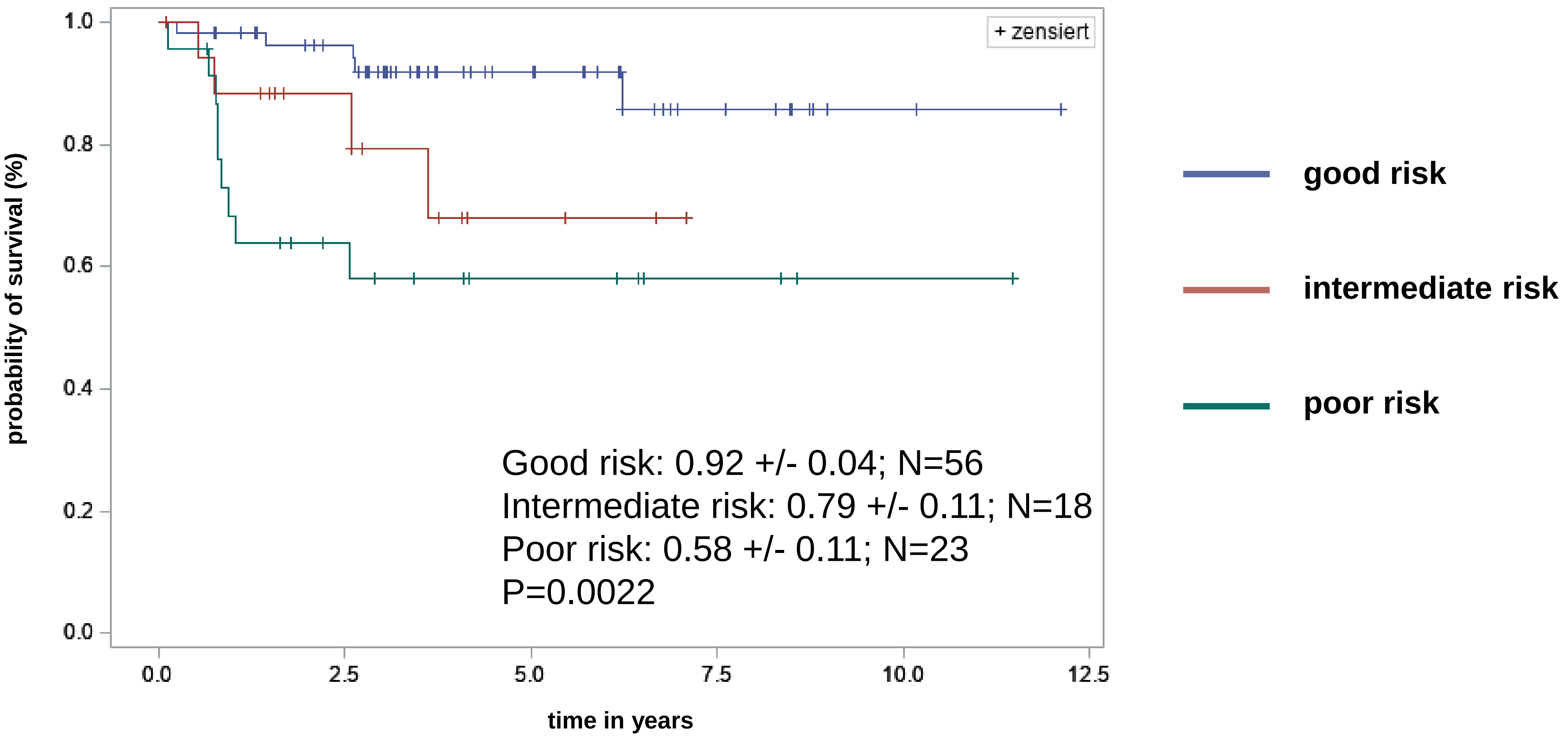

Supplementary Figure S3. Overall survival in thymic T-ALL according molecular risk groups. Like in the overall cohort, low risk comprises TLX1, NKX2-1 and LMO1 subgroups; intermediate risk HOXA, and poor risk in thymic T-ALL comprises LYL1/LMO2, TLX1 and TAL1/LMO.

T-ALL molecular subgroups (mutation rate)										
Gene	total n=83		TLX1 21	TAL1/LMO 13	HOXA 12	LYL1/LMO2 12	TLX3 10	LMO1 9	other 6
	Count	Frequency	n (%)	n (%)	n (%)	n (%)	n (%)	n (%)	n (%)
**NOTCH1**	42	51%	15 (71%)	6 (46%)	4 (33%)	5 (42%)	5 (50%)	4 (44%)	0 (0%)
**PHF6**	31	37%	14 (67%)	2 (15%)	3 (25%)	2 (17%)	4 (40%)	1 (11%)	1 (17%)
**DNM2**	15	18%	3 (14%)	0 (0%)	2 (17%)	5 (42%)	2 (20%)	1 (11%)	2 (33%)
**PTEN**	15	18%	3 (14%)	4 (31%)	1 (8%)	1 (8%)	2 (20%)	2 (22%)	3 (50%)
**FBXW7**	13	16%	3 (14%)	2 (15%)	1 (8%)	3 (25%)	2 (20%)	2 (22%)	0 (0%)
**JAK3**	12	14%	3 (14%)	1 (8%)	2 (17%)	1 (8%)	3 (30%)	1 (11%)	2 (33%)
**BCL11B**	8	10%	3 (14%)	0 (0%)	1 (8%)	1 (8%)	0 (0%)	1 (11%)	1 (17%)
**FAT1**	8	10%	3 (14%)	0 (0%)	0 (0%)	0 (0%)	1 (10%)	1 (11%)	0 (0%)
**KMT2D**	8	10%	1 (5%)	2 (15%)	1 (8%)	3 (25%)	1 (10%)	0 (0%)	0 (0%)
**NRAS**	8	10%	3 (14%)	0 (0%)	0 (0%)	2 (17%)	1 (10%)	0 (0%)	0 (0%)
**SUZ12**	8	10%	2 (10%)	0 (0%)	0 (0%)	1 (8%)	4 (40%)	0 (0%)	1 (17%)
**JAK1**	7	8%	2 (10%)	0 (0%)	1 (8%)	2 (17%)	2 (20%)	0 (0%)	1 (17%)
**SETD2**	7	8%	3 (14%)	0 (0%)	1 (8%)	2 (17%)	0 (0%)	1 (11%)	0 (0%)
**WT1**	6	7%	0 (0%)	0 (0%)	2 (17%)	3 (25%)	1 (10%)	0 (0%)	0 (0%)
**CNOT3**	5	6%	1 (5%)	0 (0%)	1 (8%)	1 (8%)	1 (10%)	0 (0%)	1 (17%)
**DNMT3A**	5	6%	2 (10%)	0 (0%)	0 (0%)	2 (17%)	0 (0%)	0 (0%)	0 (0%)
**IL7R**	5	6%	1 (5%)	1 (8%)	0 (0%)	2 (17%)	1 (10%)	0 (0%)	0 (0%)
**EP300**	4	5%	1 (5%)	1 (8%)	0 (0%)	2 (17%)	0 (0%)	0 (0%)	0 (0%)
**TP53**	4	5%	0 (0%)	1 (8%)	1 (8%)	1 (8%)	0 (0%)	0 (0%)	0 (0%)
**KRAS**	3	4%	2 (10%)	0 (0%)	0 (0%)	0 (0%)	1 (10%)	0 (0%)	1 (17%)
**RPL5**	3	4%	2 (10%)	0 (0%)	0 (0%)	1 (8%)	0 (0%)	0 (0%)	0 (0%)
**RUNX1**	3	4%	0 (0%)	1 (8%)	1 (8%)	1 (8%)	0 (0%)	0 (0%)	0 (0%)
**SH2B3**	3	4%	0 (0%)	0 (0%)	1 (8%)	1 (8%)	1 (10%)	0 (0%)	0 (0%)
**STAT5B**	3	4%	0 (0%)	0 (0%)	1 (8%)	1 (8%)	0 (0%)	0 (0%)	0 (0%)

Supplementary Table S8. Mutations according molecular subgroups.
Mutation status for 204 leukemia associated genes in 83 adult T-ALL patients. The 22 most frequently affected genes known to be mutated in T-ALL are shown. A list of investigated genes could be found in Supplementary Table S3. The full list of all variants is depicted in Supplementary Table S9.
